# Seroprevalence and distribution of leptospirosis serovars among wet market workers in northeastern, Malaysia: a cross sectional study

**DOI:** 10.1186/s12879-018-3470-5

**Published:** 2018-11-14

**Authors:** Mas Harithulfadhli Agus Ab Rahman, Suhaily Mohd Hairon, Rukman Awang Hamat, Tengku Zetty Maztura Tengku Jamaluddin, Mohd Nazri Shafei, Norazlin Idris, Malina Osman, Surianti Sukeri, Zainudin A. Wahab, Wan Mohd Zahiruddin Wan Mohammad, Zawaha Idris, Aziah Daud

**Affiliations:** 1 Department of Community Medicine, School of Medical Sciences, Universiti Sains Malaysia, 16150 Kubang Kerian, Kelantan Malaysia; 2 Department of Medical Microbiology and Parasitology, Faculty of Medicine and Health Sciences, Universiti Putra Malaysia, UPM, 43400 Serdang, Selangor Malaysia; 3 Health Department of Federal Territory Kuala Lumpur & Putrajaya, Jalan Cenderasari, 50590 Kuala Lumpur, Malaysia; 4 Health Promotion Unit, Penang State Health Department, Floor 7, Bangunan Persekutuan, Jalan Anson, 10400 Penang, Malaysia

**Keywords:** Leptospirosis, Seroprevalence, Wet market workers, High risk group

## Abstract

**Background:**

Leptospirosis is a zoonotic disease associated with occupations which exposed workers to environments contaminated with urine of infected animals. The objective of this study was to determine the seroprevalence of leptospirosis among wet market workers in Kelantan.

**Methods:**

A cross sectional study was conducted in two main wet markets in Kelantan and 232 wet market workers were randomly selected. Blood samples were analysed for microscopic agglutination test (MAT) against 20 live leptospirosis reference serovars. MAT titres of 1:100 or more were considered as seropositive.

**Results:**

It was found that the overall seroprevalence for leptospirosis among the respondents was 33.6% (95% CI = 27.5, 39.7). The samples were tested positive against serovars *Melaka* (IMR LEP 1)*, Terengganu* (IMR LEP 115)*, Sarawak* (IMR LEP 175)*, Copenhageni* (IMR LEP 803/11)*, Hardjobovis* (IMR LEP 27)*, Australis, Autumnalis, Bataviae, Canicola, Grippotyphosa, Hardjoprajitno, Icterohaemorrhagiae, Javanica, Pyrogenes, Terrasovi, Djasiman, Patoc* and *Pomona.* The predominant serovars was *Autumnalis* (18.2%).

**Conclusion:**

Wet markets workers were at risk for leptospirosis infection evidenced by high seroprevalence of leptospirosis in this study. Further research need to be conducted to determine factors that favours infection in this groups.

## Background

Leptospirosis is a worldwide zoonotic disease caused by spiral-shaped bacteria of the genus *Leptospira* which can be categorized into pathogenic and saprophytic groups. The pathogenic groups caused disease in human and become the concern of health authority. More than 250 *Leptospira* serovars have been recognized all over the world [1]*. In Malaysia, 37 serovars of Leptospira have been identified from human and animal samples* [2]*.*

Human leptospirosis is acquired through contact with urine of infected animals, either directly or indirectly. Rodents were the natural reservoirs for the bacteria. Other infected animals including horses, cows, goats, pigs, cats and dogs can be carriers for the bacteria if they are not treated. These animals can excrete leptospires through urine during their lifetime and contaminate the environment. The bacteria can gain entry into the human ecosystem through cuts in skin and mucous membranes [3]. Infected humans develop a spectrum of symptoms that can mimic other febrile illnesses including dengue, malaria and typhoid [4].

Leptospirosis causes significant morbidity and mortality with an estimation of more than one million cases and 58,900 deaths worldwide on an annual basis. The majority of the cases happen in tropical and the world’s poorest regions such as South and Southeast Asia [5]. Figures on leptospirosis can potentially be higher than reported since the true extent of cases remains unknown due to difficulty in diagnosing the condition and lack of systematic surveillance [6]. In Malaysia, leptospirosis is endemic, and the number of cases showed an increasing trend over the years and is becoming a genuine public health challenge [7].

Certain groups have been recognized to have a higher risk of infection due to the increased likelihood of contact with contaminated animals or environments [4]. Leptospirosis has been recognized as a hazard in certain occupations with increased exposure to infected animal such as agricultural workers, sewage workers, military personnel, veterinary and animal handlers [8, 9].

Another potential group at risk are wet market workers. Wet markets are places where people sell fresh meat and produce. Human activities at wet markets can provide a suitable environment and a rich source of food favouring the presence of rodents. Studies on rodents and environmental samples at wet market areas showed presence of pathogenic *Leptospira species* [10–12]. Hence, the aim of this study was to determine the seroprevalence of leptospirosis among wet market workers in Kelantan, Malaysia.

## Methods

A cross sectional study was conducted in two main wet markets in Kelantan, which is in the northeastern part of Peninsular Malaysia. This study was carried out from January to June 2017. Two main wet markets selected for this study were Siti Khadijah Market in the Kota Bharu district and Pasir Mas Market in Pasir Mas district. In 2014, Kota Bharu and Pasir Mas districts had recorded the highest number of leptospirosis cases in Kelantan and these wet markets were selected as they were the largest wet markets in the areas. Systematic random sampling was used to select participants from list of wet market workers obtained from the local municipals. A total of 232 workers from those wet markets were selected in this study. Sample size was calculated using one proportion formula and *p* = 0.35 [13] was used as a reference. Workers who were at the age of 18 years or above and worked for at least three months at the wet markets were eligible for inclusion in the study. Non-citizen and workers who were not available during the study period were excluded from the study.

Before the conduct of the study, ethical clearance was obtained from Research and Ethic Committee (Human), School of Medical Sciences, Health Campus, Universiti Sains Malaysia (USM/JEPeM/15120552). The study was explained in sufficient detail and written consent was obtained from all participants. A proforma was used to gather information on sociodemographic and professional information of the participants. Venous blood samples were collected from the participants using standard procedures [14]. The blood samples were then centrifuged for 10 min at 1300 to 2000 rpm. The separated serum was kept in a plastic screw-cap vial and stored at -20 °C until MAT was performed.

A microscopic agglutination test (MAT) was carried out at the Microbiology Laboratory of Universiti Putra Malaysia following standard methods. Serum samples were serially diluted in microtiter plates and live leptospires representing 20 reference serovars were added to each well. The plates were then incubated for two hours at 30 °C and examined using dark field microscope. The mixture was considered as positive when it showed 50% agglutination leaving 50% free cells compare to the control culture. MAT titre of 1:100 or more was used as cut off point for seropositive result [4]. This cut off point had also been used in other seroprevalence studies [13, 15].

The samples were tested for leptospiral antibodies against 20 live reference serovars as recommended by the Institute for Medical Research (IMR), Malaysia. The types of *Leptospira* tested for this study were from serovars *Melaka* (IMR LEP 1)*, Terengganu* (IMR LEP 115)*, Sarawak* (IMR LEP 175)*, Copenhageni* (IMR LEP 803/11)*, Hardjobovis* (IMR LEP 27)*, Lai, Australis, Autumnalis, Bataviae, Canicola, Celledoni, Grippotyphosa, Hardjoprajitno, Icterohaemorrhagiae, Javanica, Pyrogenes, Terrasovi, Djasiman, Patoc* and *Pomona* [16].

Data were analysed using IBM SPSS statistics version 24.0. Numerical variables were presented as means and standard deviations (SD) whereas categorical data were presented as frequencies and percentages. The seroprevalence was calculated by dividing number of positive MAT over total samples and presented as percentage with 95% confidence interval (CI).

## Results

A total of 232 wet market workers participated in this study. Table 1 shows the characteristics of the participants. All of them were Malays. The mean (SD) age was 42.6 (14.7) years old with the age range of 18 to 79 years old. Majority of them were female and 77.2% were married. Regarding level of education, more than half of the participants had at least attended secondary school. Out of 232 blood samples tested for antibodies against leptospirosis, 78 samples were positive, defining the overall seroprevalence of leptospirosis among wet market workers at 33.6% (95% CI = 27.5, 39.7). Both markets had similar results with 39 out of 116 workers were tested positive (33.6%).

**Table 1:** Socio-demographic characteristics of respondents (*n* = 232)

Variables	Frequency (%)		
	Overall *n* = 232	Siti Khadijah market *n* = 116	Pasir mas market *n* = 116
Age	42.6 (14.7) ^a^	42.0 (15.5)^a^	43.0 (13.8)^a^
Gender			
Male	83 (35.8)	34 (29.3)	51 (44.0)
Female	149 (64.2)	82 (70.7)	65 (56.0)
Ethnicity			
Malay	232 (100)	116 (100)	116 (100)
Marital Status			
Single/widower	53 (19.0)	30 (25.8)	23 (19.8)
Married	179 (77.2)	86 (74.2)	93 (80.2)
Educational Level			
No formal education	19 (8.2)	8 (6.9)	11 (9.5)
Primary school	30 (12.9)	14 (12.1)	16 (13.8)
Secondary school	137 (59.1)	64 (55.2)	73 (62.9)
Form 6/Diploma/Others	46 (19.8)	30 (25.9)	16 (13.8)

^a^Mean (SD)

Table 2 shows the distribution of positive leptospirosis serovars tested in this study. Respondents in this study can be positive to more than one leptospiral serovars. Out of 20 live reference serovars used for MAT analysis, 18 serovars were tested positive to at least one serum sample. Serovars *Autumnalis, Sarawak* (IMR LEP 175) and *Copenhageni* (IMR LEP 803/11) were the most common serovars to be positive by MAT which contributed more than one third of positive MAT results. No samples were positive for serovars *Lai* and *Celledoni*.

**Table 2:** Serovars distribution among positive MAT results on all serovars (*n* = 137)

Serovars	Overall		Siti Khadijah market		Pasir mas market	
	Frequency (*n* = 137)	Percentage (%)	Frequency (*n* = 68)	Percentage (%)	Frequency (*n* = 69)	Percentage (%)
Autumnalis	25	18.2	8	11.8	17	24.6
Sarawak (IMR LEP 175)	21	15.4	13	19.1	8	11.6
Copenhageni (IMR LEP 803/11)	12	8.8	8	11.8	4	5.8
Canicola	10	7.3	8	11.8	2	2.9
Djasiman	9	6.6	5	7.4	4	5.8
Australis	8	5.8	2	2.9	6	8.7
Patoc	8	5.8	3	4.4	5	7.2
Hardjoprajitno	7	5.1	1	1.5	6	8.7
Pyrogenes	7	5.1	4	5.8	3	4.3
Tarassovi	6	4.4	6	8.8	0	0.0
Pomona	6	4.4	4	5.8	2	2.9
Javanica	5	3.6	1	1.5	4	5.8
Icterohaemorrhagiae	4	2.9	0	0.0	4	5.8
Grippotyphosa	3	2.2	2	2.9	1	1.5
Hardjobovis (IMR LEP 27)	2	1.5	1	1.5	1	1.5
Bataviae	2	1.5	0	0.0	2	2.9
Melaka (IMR LEP 1)	1	0.7	1	1.5	0	0.0
Terengganu (IMR LEP 115)	1	0.7	1	1.5	0	0.0

Respondents can be positive to more than one serovars

Total of 137 positive MAT results on all serovars

Table 3 shows the MAT results according to products sold by the participants. The highest seroprevalence was noted in participants who sold processed food (36.4%). It was followed by other products (34.7%), fresh meat and fish (31.6%) and fruits and vegetables (25.7%).

**Table 3:** Seroprevalence of leptospirosis according types of product sold (*n* = 232)

Type of product	No. of workers	MAT 1 ≥ 100	
		Frequency (%)	95% CI
Processed food	77	28 (36.4)	25.7, 48.1
Fruits and vegetables	35	9 (25.7)	12.5, 43.3
Fresh meat and fish	19	6 (31.6)	12.6, 56.6
Others (kitchen utensils, toys)	101	35 (34.7)	25.5, 44.8

## Discussion

Leptospirosis has long been associated with occupations and activities that increase interactions between humans, animals and contaminated environments. Both wild and domestic animals can spread *Leptospira* through their urine. However, most human infections have been attributed to rodents [2]. Environments at wet market areas have provided a favourable condition for rodents’ infestation due to availability of foods and breeding place. Unhygienic conditions, open sewers and poor drainage at wet markets further attract these pests to populate the areas. The wet and moist conditions which are common in wet markets are also suitable for survival of *Leptospira* where they can survive for several weeks in stagnant water and wet soil [17].

The result of this study showed high seroprevalence of leptospirosis among wet market workers in Kelantan. This outcome supports the suspicion on risk of leptospirosis to wet market workers as they are exposed to contaminated environments and rodents at their workplace. These are supported by previous studies which noted the presence of pathogenic *Leptospira spp* in soil and water samples taken from wet markets areas [10, 11]. Azali et al. (2016) reported that 19.4% water samples taken from market areas were positive for *Leptospira spp* [10] whereas Benacer et al (2013) found that 23.1% of water and 23.3% of soil cultures were positive for *Leptospira spp* [11]. Study on rodents from wet market areas in Kuala Lumpur by Benacer et al. (2013) also noted the presence of pathogenic *Leptospira spp* in the animal samples [12].

Studies on high risk groups showed a wide range of seroprevalence for leptospirosis. This indicates different degrees of exposure to contaminated environments and animals. Shafei et al. (2012) reported overall seroprevalence of 24.7% among town service workers in a study done in Kota Bharu, Kelantan [15]. The seroprevalence varies according to job categories which were garbage collector (27.4%), landscaper (23.8%), town cleaner (26.0%) and lorry driver (17.9%). Work activities and duration of exposure to contaminated environments have contribute to high seroprevalence among garbage collectors. Another study which was conducted among 350 high-risk palm oil planters in the southern state of Malaysia showed an overall seroprevalence of 28.6%. The seroprevalence ranged from 21.1 to 59.2% between different job categories. Fruit collectors, harvesters and pesticide applicators were job categories with the highest seroprevalence due to longer contact with polluted soil and water [18]. In our study, the seroprevalence varies from 25.7 to 36.4% between workers and their type of product sold. Workers who sell processed food had the highest seroprevalence. This can be due to availability of food that were stored in their shop which can lead to rodents’ infestation. These workers shared similar workplace in a confined area and rodents can move and excrete the bacteria through their urine into the environment at the wet market. This factor might explain the seroprevalence level of workers who sell fruits, vegetables, fresh meat, fish and other products.

The predominant serovars detected in this study were *Autumnalis, Sarawak* (IMR LEP 175) and *Copenhageni* (IMR LEP 803/11) which constituted more than 40% of the immune responses among the participants. Out of 20 serovars tested, 18 serovars were tested positive with the blood samples from wet market workers. Other studies among high risk groups in Malaysia demonstrated several types of serovars detected. Ridzuan et al. (2016) reported 9 types of serovars tested positive with samples from high risk planters with the *Sarawak* (IMR LEP 175)*, Patoc* and *Celledoni* were found to be the predominant serovars [18]. A study among town service workers by Shafei et al. (2012) noted 12 types of serovars were positive with the *Patoc, Bataviae* and *Javanica* were predominant [15]. A review on leptospirosis cases in Malaysia noted about 37 serovars had been isolated from animals and human. The wet and warm climate in this region provides suitable conditions for *Leptospira* to be firmly established in the diverse environments [2].

## Conclusion

The findings in this study demonstrates high seroprevalence of leptospirosis among wet market workers in Kelantan. As wet markets are places where buyers and sellers meet, the risk of infection may also be shared by the public. Thus, further assessment should be carried out to determine factors that contribute to increase risk to leptospirosis infection in wet market places which will assist in the development of preventive measures for workers as well as to the public.

## Abbreviations

IMR: Institute for Medical Research
MAT: Microscopic agglutination test
